# Graphites *vs.* Cicatricial Tissue

**Published:** 1892-12

**Authors:** Caroline E. Hastings

**Affiliations:** Boston, Mass.


### GRAPHITES *vs.* CICATRICIAL TISSUE.

CAROLINE E. HASTINGS, M. D., BOSTON, MASS.

About two years ago Mrs. W—— came to me for consultation. She had been married about two years, and was disappointed because she had never become pregnant. A digital examination revealed what appeared to be a strong fibrous septum across the vaginal passage about an inch and a half above the vaginal orifice. To the touch it seemed complete, and a careful search with the eye after the introduction of a speculum revealed no opening. But as the patient menstruated regularly, and without suffering, I knew an opening must exist. I therefore requested my patient to come to me at the beginning of the next menstrual period. She came soon after, and I introduced a speculum and carefully wiped the surface of the septum, and watched for the manifestation of an opening by the appearance of a drop of the menstrual blood. In a moment a red point was visible, and only a point. I placed a small probe-pointed sound against the indicated opening and it passed into a chamber above the septum, and as far as I could determine into the ordinary vaginal passage. I explained the condition to my patient, and fixed upon a date for an operation. I supposed at the time that I had only a simple septum, possibly an exaggerated hymen to deal with, and that a crucial incision with the opening as a starting point would be all that would be required. Under ether the almost pin-point opening was found; dilatation made until the finger could pass, and then, instead of a smooth surface on the other side of the supposed septum, I found a mass of adhesive bands. I could make out a cervix uteri, and that the os was free.

Instead of a clean crucial incision I was obliged to carefully dissect up and free the passage above the septum, also the uterus, which had one strong band attached to the left side of the cervix. After some little effort the passage was made clear, but with a rough and bleeding surface. The result to be obtained now was healing of this surface without adhesions.

To accomplish this the surfaces were kept from contact by tampons of cotton, wet with water, with a few drops of Calendula added. The tampons were renewed every day, a douche of water and Calendula following the removal of the packing. Healing progressed favorably, and at the end of four weeks the patient was able to return to her home, but with the advice that she should come to me once a week that I might be sure matters were keeping right. At the second visit, about five weeks after the operation, I discovered a contraction of the upper portion of the vaginal passage. At the third visit, a week later, a semicircular band was very apparent. I viewed this threat of a return to the old condition with dismay, yet saw no way to prevent it. At the time of the fourth visit, or eight weeks after the operation, there was a decided cicatricial band, which to the finger, in form and feel reminded me of the falx cerebri; it was crescent-shaped and cord-like to the finger, constricting the passage and catching the lower half of a cylindrical speculum, and so preventing its introduction. Above and beyond the margin of the band I could see the os and anterior lip of the uterus. I felt that the operation had been useless, that even if pregnancy took place this band would prove a barrier to the passage of the head in parturition, and I wondered if it could be cut at the time. Then occurred to me the power of Graphites to affect cicatricial tissue, and I determined to try it. I should say that Mrs. W. was a splendidly healthy woman, and gave me no symptoms upon which to base a prescription. December 15th, 1890, I gave a dose of Graphites $^{5m}$. At the next visit the band was less resistant.

January 1st, 1891, I gave another dose, and from this time the band gradually softened, and at the end of two months had entirely disappeared, and up to the present time—more than a year—there has been no return of the contraction.

The speculum passes easily and engages the cervix without difficulty.

### Discussion.

Dr. S. Long—I should like to hear some opinion as to what would have been the effect of Graphites before the operation? I will answer my own question by saying that I believe that Graphites would have cured the case in the beginning. The doctor thought that with her surgical knowledge she could remove the difficulty with an operation, but she failed. What a blessed thing it is that she knew Homœopathy after that surgical failure. As surgeons are we justified in putting our surgical knowledge before our Homœopathy?

Dr. Hastings—If I had known in the beginning of the adhesive bands, I should probably have thought of Graphites at once. If I had given ether and dilated to the point where the finger could have been passed, before the operation, I should have discovered the bands. The origin of these bands will probably never be known. I questioned my patient carefully to find whether there was any history of injury that might have resulted in such bands, but could get no light upon it. Had it been an exaggerated hymen, I do not believe that Graphites would have removed it.

Dr. James—The late Dr. H. N. Guernsey had a case similar to that, but without the adhesive bands. He gave Silicea and cured it.

